# Microstructural White Matter Abnormalities in the Dorsal Cingulum of Adolescents with IBS

**DOI:** 10.1523/ENEURO.0354-17.2018

**Published:** 2018-08-14

**Authors:** Catherine S. Hubbard, Lino Becerra, Nicole Heinz, Allison Ludwick, Tali Rasooly, Anastasia Yendiki, Rina Wu, Neil L. Schechter, Samuel Nurko, David Borsook

**Affiliations:** 1 Center for Pain and the Brain, Boston Children’s Hospital, Waltham, MA; 2 Department of Anesthesiology, Perioperative and Pain Medicine, Boston Children’s Hospital, Boston, MA; 3 Department of Anaesthesia, Harvard Medical School, Boston, MA; 4 Athinoula A. Martinos Center for Biomedical Imaging, Massachusetts General Hospital, Charlestown, MA; 5 Department of Radiology, Harvard Medical School, Boston, MA; 6 Division of Gastroenterology, Hepatology and Nutrition, Center for Motility and Functional Gastrointestinal Disorders, Boston Children’s Hospital, Boston, MA; 7 Department of Pediatrics, Harvard Medical School, Boston, MA

**Keywords:** abdominal pain, DTI, functional GI disorders, IBS, pediatrics

## Abstract

Alterations in fractional anisotropy (FA) have been considered to reflect microstructural white matter (WM) changes in disease conditions; however, no study to date has examined WM changes using diffusion tensor imaging (DTI) in adolescents with irritable bowel syndrome (IBS). The objective of the present study was two-fold: (1) to determine whether differences in FA, and other non-FA metrics, were present in adolescents with IBS compared to healthy controls using whole-brain, region of interest (ROI)-restricted tract-based spatial statistics (TBSS) and canonical ROI DTI analyses for the cingulum bundle, and (2) to determine whether these metrics were related to clinical measures of disease duration and pain intensity in the IBS group. A total of 16 adolescents with a Rome III diagnosis of IBS (females = 12; mean age = 16.29, age range: 11.96–18.5 years) and 16 age- and gender-matched healthy controls (females = 12; mean age = 16.24; age range: 11.71–20.32 years) participated in this study. Diffusion-weighted images were acquired using a Siemens 3-T Trio Tim Syngo MRI scanner with a 32-channel head coil. The ROI-restricted TBSS and canonical ROI-based DTI analyses revealed that adolescents with IBS showed decreased FA in the right dorsal cingulum bundle compared to controls. No relationship between FA and disease severity measures was found. Microstructural WM alterations in the right dorsal cingulum bundle in adolescents with IBS may reflect a premorbid brain state or the emergence of a disease-driven process that results from complex changes in pain- and affect-related processing via spinothalamic and corticolimbic pathways.

## Significance Statement

In the present study we observed white matter (WM) abnormalities indexed by decreased fractional anisotropy (FA) in the right dorsal cingulum in adolescents with irritable bowel syndrome (IBS) compared to a healthy cohort. However, this decrease in WM FA was not related to disease severity measures. These findings represent the first report of WM brain changes in an adolescent population with IBS.

## Introduction

Irritable bowel syndrome (IBS) is a common functional gastrointestinal disorder (FGID) affecting both adults and children (Longstreth et al., 2006). Like many other chronic pain conditions, there is accumulating evidence, predominantly in adults, that this disorder affects brain structure and function (Martucci and Mackey, 2016; Weaver et al., 2016). Recurring pain associated with other symptoms (e.g., alterations in bowel habits, affect) is present in IBS and may precede or drive the observed brain changes. In children, these changes may have both early and long-term effects on behavior and disease comorbidity (viz., long-term vulnerability to psychiatric illness including anxiety, depression, and somatoform disorders; Chitkara et al., 2008; Bradford et al., 2012; Surdea-Blaga et al., 2012; Shelby et al., 2013).

The cingulate cortex is considered a key hub in the putative salience and central autonomic networks, subserving a myriad of functions related to pain processing and integration, including salience detection, self-referential, and empathetic responses to pain, motor control related to pain expression and aversion, as well as the regulation of homeostatic and viscerosensory functions (Vogt et al., 1996; Bonaz, 2003; Rapps et al., 2008; Elsenbruch, 2011; Weaver et al., 2016). In adults with IBS, previous neuroimaging studies have demonstrated aberrant brain responses to actual or anticipated rectal distension in the cingulate cortex of these patients (Silverman et al., 1997; Mertz et al., 2000; Naliboff et al., 2001; Ringel et al., 2003; Verne et al., 2003; Kwan et al., 2005; Berman et al., 2008; Elsenbruch et al., 2010; Hall et al., 2010; Tillisch et al., 2011; Larsson et al., 2012). In addition, a number of studies using morphometric neuroimaging techniques have shown marked structural changes in cingulate gray and white matter (WM) architecture in adults with IBS compared to healthy individuals, including but not limited to alterations in cortical thickness, gray matter volume, and fractional anisotropy (FA; Davis et al., 2008; Taylor et al., 2009; Blankstein et al., 2010; Seminowicz et al., 2010; Chen et al., 2011; Ellingson et al., 2013; Piché et al., 2013; Hong et al., 2014). However, few studies have evaluated changes in brain structure and function in children with IBS (Huang et al., 2016; Hubbard et al., 2016; Liu et al., 2016), and no study to date has investigated differences in WM morphology in a pediatric population.

While adult studies confirm the nature of how IBS may affect the brain, evaluation of pediatric patients may confer insights into the magnitude of early life onset brain changes (related to disease duration), commonality of these changes when compared with adults and those regions of the brain that may be more or less affected during the childhood years. Here, we evaluate WM abnormalities using diffusion tensor imaging (DTI). Specifically, the aim of the current study was to examine WM changes, indexed by mean FA and other non-FA metrics [i.e., mean diffusivity (MD); radial diffusivity (RD)] in adolescents with IBS compared to healthy, age- and gender-matched controls using whole-brain, region of interest (ROI)-restricted tract-based spatial statistics (TBSS) and canonical ROI-based DTI analyses for the cingulum bundle. We chose the cingulum bundle for our ROI analyses given its key role in processing the affective-motivational and cognitive-evaluative components of pain, integration of homeostatic-viscerosensory and motor functions, as well as recent findings demonstrating aberrant functional connectivity patterns between this region and intrinsic brain networks during liminal and subliminal rectal distension in pediatric IBS subjects (Derbyshire, 2003; Liu et al., 2016; Weaver et al., 2016). We hypothesized that specific WM abnormalities localized to the cingulum bundle would be evident in adolescents with IBS. It was also predicted that early changes in diffusion derived metrics for the cingulum bundle would be related to symptom severity measures such as disease duration and pain intensity. Lastly, our rationale for conducting ROI analyses using two different approaches was based on studies reporting high variability in DTI parameters extracted from small structures and the possibility of including voxels impacted by partial volume effects (Ozturk et al., 2008; Hakulinen et al., 2012; Lilja et al., 2016). Given the ease of which TBSS can be performed, combined with an improved ability to detect small changes in FA between groups in an unbiased manner (Smith et al., 2006), and a previous report indicating high test-retest reliability and low variability (Lilja et al., 2016), we use this as our primary method and compare these results with the canonical ROI-based DTI approach.

## Materials and Methods

### Participants

Nineteen adolescents [16.00 ± 2.09 years (mean ± SD); females = 15] with a diagnosis of IBS were prospectively recruited and enrolled in the current study. Human subjects were recruited from the Motility and Functional Gastrointestinal Disorders Center and the General GI Clinics at Boston Children's Hospital. Patients were frequency matched for age and gender to a sample of 19 healthy controls taken from our existing imaging database. General inclusion criteria required that all participants were right-handed, between the ages of 8 and 21 years, had no indication of claustrophobia or suicidal ideation, passed MRI safety screening, tested negative for drugs of abuse in their urine, and for females, were not pregnant or planning to become pregnant. Scanning parameters for patient and control groups were identical and conducted using the same scanner. This study was conducted in accordance with the Declaration of Helsinki and approved by the Boston Children's Hospital Institutional Review Board. All participants provided written informed consent or verbal assent (with parental permission for children <18 years).

For IBS patients, diagnosis was made by a gastroenterologist using pediatric Rome III criteria (Drossman et al., 2006; Rasquin et al., 2006). Exclusion criteria for the patient group included any evidence of organic gastrointestinal (GI) disease or hepatic disorders, or a serious CNS medical condition. Proton pump inhibitors and antispasmodics were allowed for patients on a stable dose for at least a four-week period before study inclusion. Patients on a stable (more than or equal to three months), low to moderate dose of a psychotropic medications such as anti-depressants or anxiolytics were also included.

### Clinical and psychometric measures

Primary clinical severity measures included disease duration (duration of IBS symptoms in years) and pain intensity in regard to abdominal symptoms (over the past week; 0 = no pain and 10 = worst pain imaginable). The gastroenterologist assessed these measures verbally at the initial clinical visit. Before scanning patients completed a series of questionnaires including the abdominal pain index (API; Walker et al., 1997; Laird et al., 2015), the pediatric quality of life inventory (PedsQL; version 4.0; child report, ages 8–12; teen report, ages 13–18; young adult report, ages 18–25; Varni et al., 2001), the PedsQL GI symptoms module (PedsQL GI module; version 3.0; Varni et al., 2014), and the functional disability inventory (FDI; Walker and Greene, 1991). Other self-report measures included the revised children’s anxiety and depression scale (RCADS; Chorpita et al., 2000), and the pain catastrophizing scale-child version (PCS-C; Crombez et al., 2003).

### FA

DTI is a MRI technique that exploits the inherent property of water molecules to diffuse in an anisotropic manner along neuronal fiber tracts, allowing for noninvasive visualization of microstructural changes in the human brain *in vivo* (Le Bihan et al., 2001; Soares et al., 2016). This technique permits both qualitative and quantitative measurement of the magnitude, directionality and overall structural integrity of WM, using DTI-derived metrics such as FA, MD, and RD (Basser, 1995; Pierpaoli et al., 1996). FA is thought to be a global measure of WM integrity, and may reflect nonspecific changes in WM due to various pathologic processes. In contrast, MD measures the rate of diffusion within a given voxel and is related to the amount of water contained within extracellular space, therefore it is inversely related to membrane density, whereas RD may be an indicator of demyelination/dysmyelination (Alexander et al., 2007; O'Donnell and Westin, 2011).

### MRI data acquisition

Diffusion-weighted images (DWIs) were acquired using a Siemens 3-T TrioTim Syngo MRI scanner (Siemens Medical Solutions) with a 32-channel head coil. A simultaneous multi-slice generalized autocalibrating partially parallel acquisition (GRAPPA; Griswold et al., 2002; Skare et al., 2007) echo planar imaging sequence (70 axial slices, FOV = 220 mm^2^, matrix = 110 × 110, slice thickness = 2 mm, resolution = 2 mm^3^ isotropic, TR = 4600 ms, TE = 89 ms) with 64 direction diffusion gradients (*b* = 1000 s/mm^2^) was acquired with a GRAPPA acceleration factor of R = 2 and a single nondiffusion weighted volume (*b_0_* = 0 s/mm^2^).

### DTI preprocessing pipeline

DWIs were converted from dicom to nifti format using MRIConvert (version 2.0.8; https://lcni.uoregon.edu/downloads/mriconvert) and preprocessed using the functional magnetic resonance imaging of the brain (FMRIB) software library (FSL), version 5.0.6. DWI images were visually inspected and corrected for image distortions and motion artifacts using FSL’s (FSL 5.0.6; http://fsl.fmrib.ox.ac.uk/fsl/fslwiki/) eddy current correction. Individual brain masks were created with FSL’s brain extraction tool (BET) and the FMRIB diffusion toolbox (FDT; version 3.0) was used to fit a diffusion tensor model to the data for each voxel using DTIfit. FA values were calculated and fed into the voxelwise whole-brain and ROI-restricted TBSS analysis described below, followed by a canonical ROI-based DTI analysis (Smith et al., 2006, 2007).

### Statistical analysis for demographic, clinical, and psychometric measures

All statistical analyses for demographic, clinical and psychometric measures were performed in SPSS version 25. To examine whether groups differed in age, a univariate ANOVA was performed. A χ^2^ test of independence was also conducted to examine whether groups differed proportionally for the sex variable. Differences in both age and gender were not expected since groups were frequency matched on both variables. In addition, Pearson correlation coefficients were computed to investigate the relationship between the clinical and psychometric measures in the patient group. In the case where assumption of normality was not met, as determined by the Shapiro–Wilk test, scores were transformed using the natural logarithm. The resulting log-transformed scores were then used in the correlation analysis (two-tailed; *p* < 0.001 deemed significant following Bonferroni adjustment for multiple comparisons). To visualize the relationship between clinical and psychometric variables, a heat map was generated with the correlation coefficients using R statistical software program (version 3.3.3).

### Whole-brain, ROI-restricted TBSS and canonical ROI-based DTI analyses

A series of standard voxelwise analyses for whole-brain and ROI-restricted TBSS were performed in FSL (Smith et al., 2006, 2007). The FMRIB58 FA 1 mm standard space template image was used as the registration target for each subject’s FA image. Subjects’ FA images were aligned into standard 1 × 1 × 1 mm MNI152 space using the nonlinear registration tool FNIRT, merged into a single 4D image, and the mean FA volume was calculated and thinned to generate the mean FA skeleton. The mean FA skeleton was thresholded at 0.2. Individual subjects aligned FA images were projected onto this skeleton and the resulting skeletonized FA data were fed into the voxelwise nonparametric permutation test. MD and RD maps were created by applying the same transformation matrices used to obtain the FA maps.

**Figure 1.**
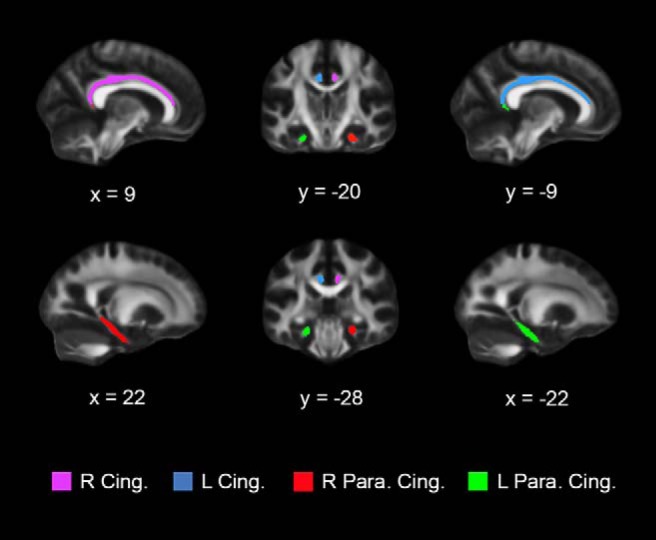
JHU WM tract masks used for the ROI-restricted TBSS and canonical ROI-based DTI analyses. Each ROI is color coded and overlaid onto a FMRIB58 FA 1 mm brain template. R Cing. = right cingulum; L Cing. = left cingulum; R Para. Cing. = right parahippocampal cingulum; L Para. Cing. = left parahippocampal cingulum.

Whole-brain and ROI-restricted TBSS analyses were performed to investigate group differences in FA using randomise, a nonparametric permutation-based inference tool for thresholding statistical maps (Winkler et al., 2014). The number of permutations was set to 10,000 and the threshold-free cluster enhancement (TFCE) option to correct for multiple comparisons were chosen (Smith and Nichols, 2009). For the ROI-restricted TBSS analysis, the mask option was also specified. Masks used for the ROI-restricted TBSS analyses were created by multiplying the mean FA skeleton mask, which was binarized and thresholded at 0.95 (i.e., *p* = 0.05), with each of the four ROIs (Fig. 1) using fslmaths. This resulted in the following four masks: right cingulum mask (voxels = 425), left cingulum mask (voxels = 469), right parahippocampal cingulum mask (voxels = 421), and left parahippocampal cingulum mask (voxels = 442). ROIs for the cingulum were obtained directly from the ICBM-DTI-81 1 mm FA atlas (Mori et al., 2008) of the Johns Hopkins University (JHU) DTI-based white-matter tractography atlas in FSL and consisted of the right and left subgenual and retrosplenial subdivisions combined, collectively referred to as the dorsal cingulum bundle, and a second pair of ROIs, containing the right and left ventral aspect of the cingulum, referred to here as the parahippocampal cingulum bundle (Fig. 1).

Voxelwise statistics on skeletonized FA and non-FA data (MD and RD) were conducted between groups using a series of two-sample t-tests with age and gender specified as covariates of no interest and the mask option flagged with each of the four aforementioned masks (i.e., right and left cingulum and parahippocampal cingulum masks) using randomise. The resulting significant TFCE-corrected *p* value statistical maps were thresholded at 0.95 (i.e., *p* = 0.05) and binarized to create masks used for extraction of mean FA values from significant clusters using fslmeants command with the 4D skeletonized FA image specified as the input. In addition, mean MD and RD values were extracted using the significant FA cluster mask and the 4D skeletonized MD or RD image specified as the input. Extracted mean FA, MD, and RD values from significant cluster masks were then imported to SPSS (version 25) and bar graphs were created to visualize differences in group means.

For the canonical ROI-based DTI analysis a semi-automated ROI definition and deprojection method was used in FSL. All subjects’ native T1 images were registered to the MNI T1 1 mm brain template. Next, each subject’s native FA image was nonlinearly registered to the MNI FMRIB58 FA 1 mm template using the fsl_reg command. Inverse nonlinear registration transformation parameters were generated and each of the four ROIs taken from the JHU ICBM-DTI-81 1 mm FA atlas (R and L dorsal cingulum and parahippocampal cingulum ROIs; Fig. 1) were deprojected using the invwarp command. Once each ROI was deprojected onto each subject’s native FA map, fslmaths was used to extract mean FA values. The same procedure was followed to obtain mean MD and RD values. Extracted mean FA, MD and RD values from the four ROIs for each subject were imported to SPSS for further analysis using a series of univariate ANOVAs with age and sex specified as covariates of no interest.

### Correlations between DTI metrics, clinical, and psychometric measures

Linear regression was performed in SPSS to examine the relationship between mean FA, MD, and RD values extracted from significant clusters and clinical measures of disease severity (i.e., disease duration and pain intensity) while controlling for age. Given the expected direction for the linear relationship between FA and disease severity measures, one-tailed tests were used for these analyses. In addition, regression analyses controlling for age were also conducted between mean FA, MD, and RD values extracted from significant clusters and scores from the psychometric measures. However, no a priori hypotheses were made regarding the expected direction; therefore, two-tailed tests were used. All *p* values were corrected for multiple comparisons using Bonferroni adjustment.

## Results

### Demographic, clinical, and psychometric measures

Of the 19 adolescents with IBS enrolled, three were excluded from all statistical analyses; one patient was excluded due to being asymptomatic at the time of scanning, while two other patients were excluded due to either missing DWI data or venetian blind-like distortions, likely a result of excessive motion. After excluding these patients, our final patient sample consisted of 16 adolescents with a Rome III diagnosis of IBS (females = 12; mean age ± SD = 16.29 ± 1.78, age range: 11.96–18.5 years). These patients were frequency matched to 16 healthy participants (females = 12; mean age ± SD = 16.24 ± 1.89; age range: 11.71–20.32 years) on age and sex variables. Table 1 summarizes patient characteristics, and Table 2 displays descriptive statistics for clinical and psychometric measures for the patient group. With regard to bowel habit subtype, the majority of patients reported constipation predominant symptoms (IBS with constipation, IBS-C = 9, IBS with diarrhea, IBS-D = 4, mixed IBS, IBS-M = 2, unspecified IBS = 1; Table 1).

**Table 1.** Patient characteristics

	Age (years)	Sex	Bowel habit	Current medications	Comorbid conditions	Disease duration	Pain intensity	API	FDI	PedsQL	GI PedsQL	T. anxiety scr	T. anxiety depress. scr	Depress. scr	PSC-C
1	16.43	F	IBS-C	Antiemetic, antispasmodic, laxative, PPI	-	3.0	2.08	2.45	7	-	-	16	23	48	-
2	16.90	F	IBS-D	None	ACNES	1.5	1.95	1.78	15	-	-	-	-	-	-
3	13.28	F	IBS-C	Antiemetic, PPI	-	2.0	1.95	2.15	30	51.09	48.31	24	37	63	3.22
4	17.59	M	IBS-D	Antiemetic, antihistamine, antispasmodic, stool softener	-	5.0	1.95	1.78	22	76.09	68.24	9	15	47	2.77
5	17.07	M	IBS-D	Antacid, antiemetic	-	3.5	2.20	2.48	38	36.96	53.38	42	50	52	3.74
6	17.55	F	IBS-C	GCC agonist, PPI	-	1.0	1.95	3.30	14	60.87	50.68	21	39	66	-
7	16.46	F	IBS-C	Antidepressant (SSRI)	ACNES	2.0	2.08	3.30	14	63.04	46.28	31	43	56	3.56
8	18.50	F	IBS-U	Antacid, antihistamine, β-blocker, NSAID, opioid analgesic (meperidine), sedative (ambien), TCA	-	2.0	2.08	3.80	30	50.00	54.0	36	52	71	3.74
9	15.86	F	IBS-C	Antihistamine, NSAID, PPI, antidepressant (TCA), levothyroxine (TH)	Ehlers-Danlos, arthritis	6.0	2.08	3.28	43	35.86	38.17	60	82	81	3.09
10	18.30	F	IBS-C	GCC agonist, PPI, muscle relaxant, anti-inflammatory	-	6.0	2.30	3.00	10	64.13	37.16	15	23	47	2.64
11	16.67	F	IBS-M	Anti-inflammatory, β-blocker, PPI, SRA (triptan), TCA	Possible POTS, occasional migraine	5.0	2.08	2.53	30	63.04	57.77	7	18	54	2.89
12	16.87	M	IBS-D	Antihistamine, PPI	-	3.0	2.20	2.60	10	80.43	75.34	12	22	57	2.89
13	15.06	F	IBS-M	Antispasmodic, laxative	-	1.0	2.20	2.88	21	-	-	8	11	35	-
14	14.65	F	IBS-C	Antidepressant (SNRI), laxative	-	7.0	2.20	3.80	12	60.87	62.50	25	39	67	3.22
15	11.96	F	IBS-C	Inhaled, corticosteroid, laxative, PPI, stool softener, TCA	-	8.0	2.08	1.65	24	52.17	64.58	24	32	8	3.43
16	17.50	M	IBS-C	Antihistamine, laxative, PPI	Barrett’s esophagus	10.0	2.08	1.35	21	81.51	60.47	23	30	49	2.77

ACNES = anterior cutaneous nerve entrapment syndrome; GCC = guanylate cyclase C agonist; NSAID = non-steroidal anti-inflammatory drug; SNRI = selective norepinephrine reuptake inhibitor; SSRI = selective-serotonin reuptake inhibitor; SRA = serotonin receptor agonist; TCA = tricyclic antidepressant; TH = thyroid hormone; PPI = proton-pump inhibitor; POTS = postural orthostatic tachycardia syndrome; API = abdominal pain index; FDI = functional; disability inventory; PedsQL = pediatric quality of life inventory; PedsQL GI = pediatric quality of life inventory gastrointestinal symptoms module; T. anxiety scr = total anxiety score; T. anxiety depress. scr = total anxiety depression score; Depress. scr = Depression score; PCS-C pain catastrophizing scale– child version.

**Table 2.** Descriptive statistics for clinical and psychometric measures

	IBS patients		
	Mean	SD	*n*
Disease duration (years)	4.125	2.699	16
Pain intensity	8.125	0.885	16
API	2.633	0.757	16
FDI	21.313	10.575	16
PedsQL (total score)	59.698	14.517	13
GI PedsQL (total score)	55.148	11.250	13
RCADS - total anxiety score	23.533	14.342	15
RCADS - total anxiety depression score	34.400	18.074	15
RCADS - depression score	53.400	17.029	15
PCS-C	25.333	10.012	12

*n* = sample size; API = abdominal pain index; FDI = functional; disability inventory; PedsQL = pediatric quality of life inventory; PedsQL GI = pediatric quality of life inventory gastrointestinal symptoms module; PCS-C = pain catastrophizing scale – child version; RCADS = revised children's anxiety and depression scale; SD = standard deviation.

A one-way ANOVA reveled no significant group differences for age [*F*_(1,31)_ = 0.005, *p* = 0.94]. The χ^2^ test (Fisher’s exact test) also showed no difference in the proportion of males to females between healthy control and IBS groups [χ^2^ (1) = 0, *p* = 1.0]. Shapiro–Wilk tests demonstrated that all variables met the assumption of normality with the exception of pain intensity, which was log-transformed, and these values were then used in all subsequent analyses.

### Whole-brain, ROI-restricted TBSS, and ROI-based DTI analyses

**Figure 2.**
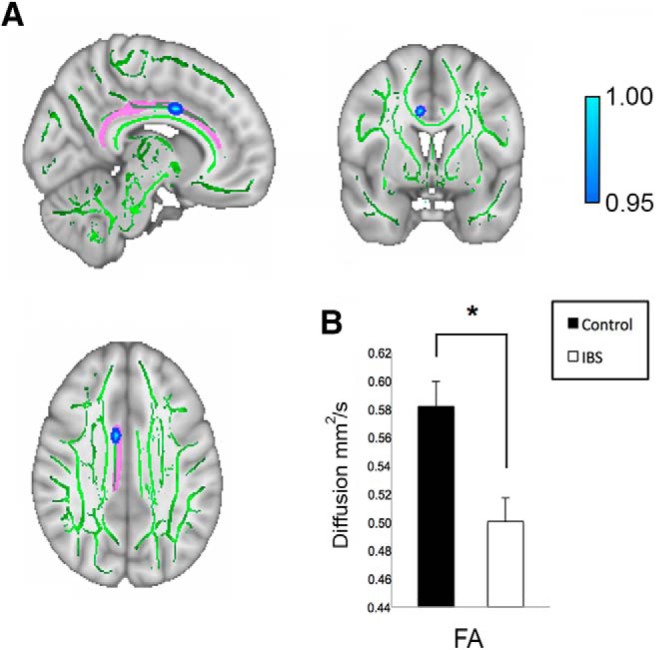
Results from the ROI-restricted TBSS group analysis for the right dorsal cingulum. ***A***, ROI-restricted TBSS analysis revealed a significant decrease in FA in adolescents with IBS compared to non-IBS cohort (IBS patients < healthy controls; *p <* 0.05, TFCE corrected; color bar is scaled from 0.95 to 1) for a cluster located in the right dorsal cingulum. TFCE-corrected map overlaid onto MNI 152 T1 1-mm brain and thresholded (0.2) mean FA skeleton (green) template in FSLView (version 3.2.0) with superimposed JHU binary mask for the right dorsal cingulum shown in pink. To improve visualization, FSL’s tbss-fill command was used to thicken tracts that showed significant group differences in FA. MNI coordinates for the peak voxel in the FA cluster are *x* = 8.83, *y* = 3.5, *z* = 33.6. Images are displayed in radiologic orientation. ***B***, Bar graph showing mean values extracted from significant FA cluster in IBS patients and healthy controls, *F*_(1,28)_ = 12.22, *p* = 0.002. Asterisk denotes significant group differences with IBS patients showing decreased FA compared to healthy controls.

**Figure 3.**
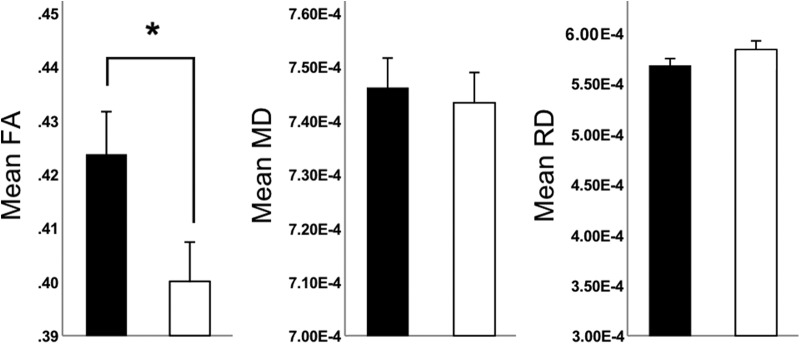
Results derived from the canonical ROI-based DTI analysis for the right dorsal cingulum. Bar graphs showing mean FA, MD, and RD values extracted from the right dorsal cingulum ROI in IBS patients (white bars) versus healthy controls (black bars). Asterisk denotes significant group differences with IBS patients showing decreased FA compared to healthy controls for the right dorsal cingulum (*p* = 0.028).

Whole-brain TBSS analysis revealed no significant group differences in mean FA or non-FA metrics (MD or RD) for adolescents with IBS versus controls following TFCE correction. In addition, we performed separate ROI-restricted TBSS analyses to examine WM FA differences for the right and left dorsal and parahippocampal cingulum in IBS patients versus controls. Results revealed a significant group difference in mean FA for the right dorsal cingulum (*p* < 0.05, TFCE corrected), but not for the left dorsal cingulum or the right or left parahpippocampal cingulum. Specifically, adolescents with IBS displayed lower FA (Fig. 2) for fiber tracts in the right dorsal cingulum compared to controls. ROI-restricted TBSS analyses performed using the right or left parahippocampal cingulum ROIs yielded no significant group differences for MD or RD following TFCE-correction. Consistent with results obtained for the ROI-restricted TBSS analysis, the ROI-based DTI analysis showed group differences in mean FA for the right dorsal cingulum bundle (*F*_(1,32)_ = 5.35, *p* = 0.028) with IBS patients exhibiting statistically significant decreased mean FA compared to healthy controls (Fig. 3); No group differences for mean MD (*F*_(1,32)_ = 0.92, *p* = 0.514) or RD (*F*_(1,32)_ = 4.25, *p* = 0.287) for this ROI, or extracted mean DTI metrics from the other ROIs were found (Table 3).

**Table 3.** Descriptive statistics for mean FA, MD, and RD extracted from the right dorsal cingulum ROI for IBS and healthy control groups for the ROI-restricted TBSS and ROI-based DTI analyses

		IBS (*n* = 16)		Controls (*n* = 16)	
Method	DTI Metric	Mean	SD	Mean	SD
ROI-restricted TBSS	FA	0.500	0.067	0.582	0.070
	MD	6.90 × 10^-4^	5.46 × 10^-5^	6.90 × 10^-4^	5.64 × 10^-5^
	RD	4.85 × 10^-4^	6.44 × 10^-5^	4.35 × 10^-4^	6.65 × 10^-5^
Canonical ROI-based DTI	FA	0.400	0.029	0.424	0.032
	MD	7.56 × 10^-4^	2.90 × 10^-5^	7.53 × 10^-4^	2.23 × 10^-5^
	RD	5.84 × 10^-4^	3.54 × 10^-5^	5.68 × 10^-4^	2.86 × 10^-5^

ROI = region of interest; FA = fractional anisotropy; MD = mean diffusivity; RD = radial diffusivity; *n* = sample size; SD = standard deviation.

To determine whether mean values for DTI metrics (FA, MD, and RD) extracted from the right dorsal cingulum differed within group using the two ROI methods (skeleton and canonical ROI analysis), a series of pairwise comparisons were conducted using paired sample t-tests, corrected for multiple comparisons with Bonferroni adjustments. Results showed that for the IBS group, mean FA [*t*_(15)_ = 7.24, *p* < 0.0001], MD [*t*_(15)_ = -5.01, *p* < 0.0001], and RD [*t*_(15)_ = -6.71, *p* < 0.0001], differed significantly between methods. Similarly, mean FA [*t*_(15)_ = 10.59, *p* < 0.0001], MD [*t*_(15)_ = -5.16, *p* < 0.0001], and RD [*t*_(15)_ = -9.70, *p* < 0.0001] also differed significantly between ROI analytical methods for the healthy control group.

### Correlations between disease severity, psychometric measures, and dorsal cingulum FA, MD, and RD in pediatric IBS

For the patient group, regression analyses revealed no significant relationship between disease duration or pain intensity and mean FA (disease duration *R* ^2^ = 0.012; pain intensity *R* ^2^ = 0.019), MD (disease duration *R* ^2^ = 0.055; pain intensity *R* ^2^ = 0.036) or RD (disease duration *R* ^2^ = 0.038; pain intensity *R* ^2^ = 0.009) extracted from the significant FA cluster in the right dorsal cingulum from the ROI-restricted TBSS analysis. Individual regression analysis performed on mean FA, MD, and RD values extracted from the TFCE-corrected significant cluster for the right dorsal cingulum and psychometric measures revealed no significant relationships following Bonferroni adjustment. Similarly, no significant relationships emerged from the regression analyses performed on extracted mean FA (disease duration *R* ^2^ = 0.110; pain intensity *R* ^2^ = 0.067), MD (disease duration *R* ^2^ = 0.022; pain intensity *R* ^2^ = 0.049), and RD (disease duration *R* ^2^ = 0.013; pain intensity *R* ^2^ = 0.046) using the standard ROI-based method for the right dorsal cingulum and disease severity measures (disease duration and pain intensity), or for the psychometric measures following Bonferroni correction.

### Correlations between clinical and psychometric variables

**Figure 4.**
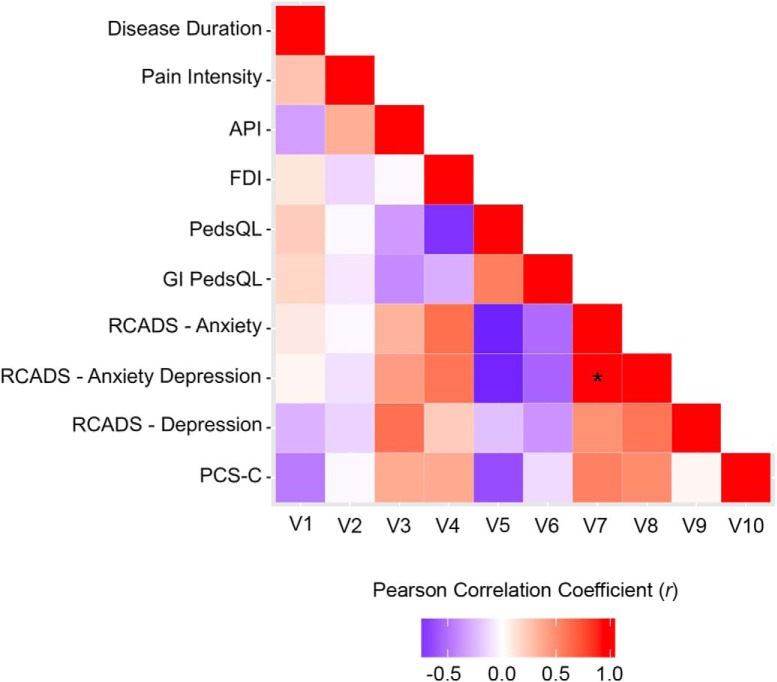
Heat map summarizing Pearson correlation coefficients (*r*) for clinical and psychometric measures, uncorrected for multiple comparisons. Asterisk denotes correlation that remained significant after Bonferroni adjustment (adjusted *p* value = 0.05/45 = 0.001).

Strength and direction of linear relationships between clinical variables and psychometric measures are displayed in a heat map of correlation coefficients in Figure 4. No significant correlations were found between disease duration or pain intensity, or between these variables and any of the psychometric measures after Bonferroni adjustment. API scores positively correlated with depression scores on the RCADS (*r* = 0.62, *p* = 0.02). FDI negatively correlated with PedsQL (*r* = -0.73, *p* = 0.005) and positively correlated with total anxiety (*r* = 0.62, *p* = 0.01) and total anxiety depression (*r* = 0.59, *p* = 0.02) RCADS scores. PedsQL positively correlated with GI PedsQL (*r* = 0.56, *p* = 0.05) and negatively correlated with total anxiety (*r* = -0.78, *p* = 0.002) and total anxiety depression (*r* = -0.77, *p* = 0.002) scores on the RCADS, and with pain catastrophizing scores from the PCS-C (*r* = -0.63, *p* = 0.03). Total anxiety and total anxiety depression scores were positively correlated (*r* = 0.98, *p* < 0.001), as was total anxiety depression scores with total depression (*r* = 0.59, *p* = 0.02). It should be noted that the aforementioned results were not corrected for multiple comparisons. Using Bonferroni adjustment (0.05/45 = 0.001), the only correlation that survived correction was between total anxiety and total anxiety depression scores (*r* = 0.98, *p* < 0.0001) from the RCADS.

## Discussion

This study represents the first report of WM abnormalities specific to the cingulum bundle in adolescents with IBS. Our ROI-restricted TBSS analysis revealed a significant group difference in mean FA that survived TFCE correction in the right dorsal cingulum. Specifically, we found decreased FA in adolescent IBS patients relative to age- and gender-matched healthy controls in the medial portion of the right dorsal cingulum bundle (i.e., mid-cingulum). Moreover, the main finding of group differences in mean FA extracted from our ROI-based DTI analysis were consistent with findings from our ROI-restricted TBSS analysis; IBS patients showed significantly lower mean FA values for the right dorsal cingulum compared to healthy controls. These results indicate that the ROI-restricted TBSS approach may be valid in restricted search areas for well-defined tracts that can be identified reliably across subjects, such as in the case of the cingulum bundle (Lilja et al., 2016). However, contrary to our predictions, clinical measures of disease duration and pain intensity were unrelated to FA in IBS patients using both ROI-based methods. Longitudinal studies are needed in larger samples of pediatric IBS patients to determine whether the WM abnormalities observed here endure over time or instead represent some sort of malleable, microstructural reorganizational changes that manifest early in the course of the disease.

### Why the cingulum?

The cingulum contains bundles of WM tracts that surround the corpus callosum, connecting the cingulate cortex with a number of limbic and paralimbic regions and cortical association areas in the frontal, temporal, and parietal lobes (Jones et al., 2013). Anatomically, it consists of many short fiber tracts that form interregional connections within the cingulum itself, and longer fiber tracts that link more distant regions together. Based on tract-tracing studies in nonhuman primates and recent tractography studies using DTI techniques in humans, the cingulum can be topographically organized into at least three subdivisions, a dorsal aspect containing the subgenual and retrosplenial subdivisions and a ventral aspect, containing the parahippocampal subdivision (Jones et al., 2013). The WM changes observed in this study were localized to an area within the retrosplenial portion of the cingulum bundle. It contains fibers that have reciprocal connections between the prefrontal cortex, anterior cingulate, and posterior cingulate cortices (Goldman-Rakic et al., 1984; Mufson and Pandya, 1984; Vogt and Pandya, 1987; Kobayashi and Amaral, 2003, 2007). In addition, there is a contribution of fiber connections from the thalamus (anterior and lateral dorsal nuclei; Mufson and Pandya, 1984). The anterior thalamus has a number of highly conserved subnuclei involved in learning and memory (Jankowski et al., 2013), which have been postulated to subserve an “extended hippocampal function” (Aggleton et al., 2010). The anterior thalamic inputs may be a conduit for the observed anatomical changes in this study. In addition to these diffuse fiber tracts that connect to thalamic regions involved in many of the maladaptive behavioral manifestations that accompany clinical presentation of IBS, there is data to support direct medial thalamic connections via the spinothalamic tract, a key pathway conveying nociceptive signals from visceral and somatic afferents (Ammons et al., 1985). Preclinical studies implicate the centromedial portion of the right and left cingulum bundle in nociception (Nikenina et al., 2008). Microinjections of lidocaine temporarily blocks formalin-induced nociceptive responses and electrical stimulation to the cingulum bundle and surrounding tissue has been shown to have analgesic effects (Vaccarino and Melzack, 1989, 1992; Fuchs et al., 1996). Moreover, the role of the cingulum in negative affective states and in the subjective experience of pain is well described. There is evidence that lesioning these fiber tracts in cases of intractable cancer pain or other chronic pain conditions results in diminished pain with patients reporting that they are less likely to be bothered by their pain (Yen et al., 2005; Sharim and Pouratian, 2016), suggesting that the cingulum may subserve evaluative functions related to the aversiveness of pain and not pain intensity per se. Extrapolating this, ongoing pain signaling via medial pain pathway may result in central sensitization of spinothalamic afferents, which over time could manifest in abnormalities further upstream, in WM tracts of the cingulum.

### WM brain changes in youth versus adults with IBS

This is the first DTI analysis in adolescents with a diagnosis of IBS. The focal changes observed in this younger population are different from the more widespread changes seen in adult IBS patients, perhaps indicative of initial effects of the disease. For example, a few studies investigating DTI related changes in adults with IBS have reported WM alterations in a number of brain regions including insula, thalamus, basal ganglia, and somatosensory cortex (Chen et al., 2011; Ellingson et al., 2013). We interpret these focal changes as likely suggestive of early expression of the disease on WM tracts; however, this may or may not be the case. A more parsimonious explanation may be that these findings simply reflect variability of the adolescent brain combined with the small sample size. Clearly, longitudinal studies are needed with larger sample sizes to provide insight into the causal relationship between early brain changes and clinical manifestations of the disease state.

FA is said to be sensitive to subtle, nonspecific changes in WM architecture indicative of neuropathological processes, whereas MD and RD may be more sensitive measures of WM pathology that results from neuroinflammation, edema, hypoxia, necrosis, tissue cellular density (i.e., cellularity), demyelination, and changes in axonal diameter or density (Sugahara et al., 1999; Gauvain et al., 2001; Beaulieu, 2002; Mac Donald et al., 2007). Although our study findings point to decreased FA in the right dorsal cingulum in young IBS patients compared to matched healthy controls, the pathogenesis underlying these WM changes cannot be discerned from the present data. WM abnormalities in FA and other non-FA metrics in the cingulum have been linked to anxiety disorders such generalized anxiety disorder, obsessive-compulsive disorder and post-traumatic stress disorder (Lochner et al., 2012; Daniels et al., 2013; Wang et al., 2016), as well a various pain conditions, including complex regional pain syndrome (CRPS), temporomandibular disorder, chronic musculoskeletal pain, trigeminal neuralgia, and IBS (Geha et al., 2008; Chen et al., 2011; Moayedi et al., 2012; Ellingson et al., 2013; DeSouza et al., 2014; Lieberman et al., 2014; Qi et al., 2016). Therefore, it is possible that the observed microstructural WM changes reflect sensitization of viscerosomatic nociceptive afferents, leading to functional reorganization in spinothalamic and corticolimbic tracts involved in processing pain and affect, including the dorsal cingulum bundle.

## Limitations

There are a number of limitations to this study: (1) Sample size: while pediatric studies are more challenging than adult studies, the small sample size and heterogeneity of bowel habit subtypes limit generalizability of our findings. (2) Medications: some of our patients were taking medications with CNS effects, including antidepressants and anxiolytics, and this may have impacted our results in an negative manner. Despite the degree of difficulty in recruiting adolescents with IBS to participate in a neuroimaging study, all effort was made to exclude patients on high doses of antidepressants or anxiolytics, and only patients on stable doses were enrolled. (3) Pubertal changes: another limitation of this study was that we did not control for pubertal changes which have been shown to be linked to brain maturation, including WM development, during childhood and adolescence (Blakemore et al., 2010; Herting and Sowell, 2017). However, we did control for age in our statistical analyses as well as age- and sex-matched subjects between groups, which should have minimized the impact of pubertal changes on our results, balancing these effects across groups. (4) Pre-existing brain state versus consequences of disease state: this is obviously difficult to know, but due to inherent genetic makeup of an individual actual manifestations of the disease state may follow these early brain changes, occurring before symptomatic disease onset. Conversely, the disease state itself may precipitate these brain changes.

## Conclusions

Our findings in young patients with IBS showed significant alterations in WM integrity for fibers in the right dorsal cingulum bundle that were not related to disease duration or abdominal pain severity. These results provide a basis for understanding the longitudinal natural history of microstructural brain changes in those patients in which the disease persists and may give some insight into the pathophysiology of IBS.

## Synthesis

Reviewing Editor: Karen Davis, Krembil Research Institute and University of Toronto Decisions are customarily a result of the Reviewing Editor and the peer reviewers coming together and discussing their recommendations until a consensus is reached. When revisions are invited, a fact-based synthesis statement explaining their decision and outlining what is needed to prepare a revision will be listed below. The following reviewer(s) agreed to reveal their identity: Massieh Moayedi.

The authors have done a tremendous job in responding to comments.

There remains 2 issues:

1) It is still not convincing that performing a voxelwise analysis in a relatively small mask is appropriate. Instead, should the authors have extracted a mean FA (or other DWI metric).

2) it is not clear whether and how the cingulum masks were merged to create a single mask. Please provide clear details, as thresholds, binarizing and overall volume are important details in assessing statistics.
